# Cost implications of delays to tuberculosis diagnosis among pulmonary tuberculosis patients in Ethiopia

**DOI:** 10.1186/1471-2458-10-173

**Published:** 2010-03-30

**Authors:** Mengiste M Mesfin, James N Newell, Richard J Madeley, Tolib N Mirzoev, Israel G Tareke, Yohannes T Kifle, Amanuel Gessessew, John D Walley

**Affiliations:** 1 Nuffield Centre for International Health and Development, Institute of Health Sciences, University of Leeds, Leeds, UK; 2 University of Nottingham Medical School, School of Community Health Science, Nottingham, UK; 3 The World Health Organization, Country Office, Addis Ababa, Ethiopia; 4 Tigray Regional Health Bureau, the Department of Diseases Prevention and control of Tigray Regional Administration, Tigray, Ethiopia; 5 Mekelle University Medical College, Tigray, Ethiopia

## Abstract

**Background:**

Delays seeking care worsen the burden of tuberculosis and cost of care for patients, families and the public health system. This study investigates costs of tuberculosis diagnosis incurred by patients, escorts and the public health system in 10 districts of Ethiopia.

**Methods:**

New pulmonary tuberculosis patients ≥ 15 years old were interviewed regarding their health care seeking behaviour at the time of diagnosis. Using a structured questionnaire patients were interviewed about the duration of delay at alternative care providers and the public health system prior to diagnosis. Costs incurred by patients, escorts and the public health system were quantified through patient interview and review of medical records.

**Results:**

Interviews were held with 537 (58%) smear positive patients and 387 (42%) smear negative pulmonary patients. Of these, 413 (45%) were female; 451 (49%) were rural residents; and the median age was 34 years. The mean (median) days elapsed for consultation at alternative care providers and public health facilities prior to tuberculosis diagnosis was 5 days (0 days) and 3 (3 days) respectively. The total median cost incurred from first consultation to diagnosis was $27 per patient (mean = $59). The median costs per patient incurred by patient, escort and the public health system were $16 (mean = $29), $3 (mean = $23) and $3 (mean = $7) respectively. The total cost per patient diagnosed was higher for women, rural residents; those who received government food for work support, patients with smear negative pulmonary tuberculosis and patients who were not screened for TB in at least one district diagnostic centers.

**Conclusions:**

The costs of tuberculosis diagnosis incurred by patients and escorts represent a significant portion of their monthly income. The costs arising from time lost in seeking care comprised a major portion of the total cost of diagnosis, and may worsen the economic position of patients and their families. Getting treatment from alternative sources and low index of suspicion public health providers were key problems contributing to increased cost of tuberculosis diagnosis. Thus, the institution of effective systems of referral, ensuring screening of suspects across the district public health system and the involvement of alternative care providers in district tuberculosis control can reduce delays and the financial burden to patients and escorts.

## Background

Ethiopia is among countries with a high burden of tuberculosis (TB). The annual incidence of smear positive pulmonary TB is still high (163 per 100,000 population) despite the implementation of Directly Observed Treatment Short course strategy (DOTS) over the past two decades [1]. This could partly be attributed to delays to TB treatment. Delay in TB diagnosis increases the risk of transmission and economic costs to patients and communities at large. The median patient delay at any public health facility is alarmingly prolonged with studies reporting from 2 to 4 months [2,3]. In Ethiopia, delays arising from the use of alternative care providers (traditional healers, private practitioners and private pharmacies) and in public health facilities constitute about 24% of the total delay [4]. Prolonged delays in accessing TB treatment have cost implications to patients, their families and the public health system [5-9]. Poverty is common among TB patients that may hinder their decision to seek early treatment [10]. Ethiopia is among the poorest countries in sub-Sahara where 65% of the population earns less than 1$ (United States Dollar) a day [11]. Increases in patient cost could therefore lead to further delays and inequity in accessing TB treatment [5]. Nevertheless, the significance of the costs incurred by patients, escorts and the public health system has never been investigated in Ethiopia.

We have recently reported our investigation about the pattern of care seeking behaviour and risk factors for delays to seek care in public health facilities among pulmonary TB patients [12]. Prolonged delay has been associated with female gender, rural poverty and illiteracy in that study. The evaluation of financial burdens encountered by patients in accessing TB care and their escorts is paramount for instituting measures for effective TB control. Here, we report the costs encountered to seek care among these patients prior to TB diagnosis. The study takes a societal perspective for evaluating these costs. The causes of delays at care providers and their cost implications were assessed in order to identify measures to reduce delays and costs of TB diagnosis within the ongoing TB control strategy.

## Methods

### Setting

A detailed description of the study setting and sampling are published in this journal http://www.biomedcentral.com/content/pdf/1471-2458-9-53.pdf. This study was carried out in 10 districts of Tigray region, northern Ethiopia. The study districts had about 1 million residents constituting 22.3% of the total regional (4.3 million) population. The main sources of health care for most people are district public health facilities which are organised into three tiers of referral: primary (clinics), secondary (health centres) and tertiary (district hospitals). Health services access, as defined by residence within 10 kilometres of any government health facility, is about 65%. Currently, the government is also introducing primary health care services in rural villages using trained health extension workers [13].

The National Tuberculosis Control Programme (NTC) is integrated within the district public health system. The DOTS programme is only available in public health facilities where patients have free access to diagnostic and treatment services. TB diagnostic services are only available in health centres and hospitals which are mainly located at district urban centres. TB diagnostic services are not available in clinics even though they are more accessible to the majority of the rural population. Health workers in clinics are therefore required to identify and refer patients who had cough for ≥ 3 weeks (*suspected cases*) to health centres [14]. Health centres have to refer those cases with negative sputum smear test for Acid Fast Bacillus to district hospitals for further investigation [14]. This system of referral of suspects among district public health facilities is anticipated to improve case detection and reduce delays to TB diagnosis. Despite the high degree of integration of DOTS programme at all levels of public health facilities, less than half of the estimated annual smear positive PTB cases are detected [15].

### Sample size and sampling procedures

All newly diagnosed pulmonary TB patients ≥ 15 years of age were prospectively assessed at the time of diagnosis from January 12, 2005 to January 12, 2006. To estimate the prevalence of patient delay in the study districts with a 95% Confidence Interval of width ± 5%, a sample size of 374 is needed, taking a prevalence of 58% patient delay from earlier Ethiopian study [4]. The sample size was increased by a factor of 2.4 to allow for drop-out and to permit reliable estimates potential risk factors for patient delay.

### Data collection

Data was collected regarding days of delay at care providers (both at alternative sources of care and public health facilities), costs incurred by patients, escorts and the public health system. Patients were interviewed using a pre-tested questionnaire translated into the local language which comprised variables for assessing time elapsed to seek care and costs incurred at each care provider prior to TB diagnosis. Patient medical records were reviewed to determine whether patients were screened for TB or were treated for other illnesses (mis-diagnosed) during their consultation at district diagnostic centres.

### Cost data

The types of cost data, costing data and sources of cost information are shown in Table 1. The ingredient approach was used to estimate costs incurred to patients, escorts and the public health system.

**Table 1:** Cost categories and methods of data collection in Tigray Region, Ethiopia.

Funding sources	Cost data and cost categories	Cost estimation	Sources of data
**Patients**	**Indirect cost**		
	Income lost for the time lost from work during consultations at various care providers.	Income lost for days way from work were estimated at local labour cost of $0.88 per day (unemployed patients) or using daily rates derived from their monthly income.	Patient interview
	Income lost for time lost from work travelling for consultations from home to public health facilities.	Income lost for travel time was estimated at labour cost of $0.11 per hour (for unemployed patients) or using hourly rates derived from their monthly incomes (for employed patients).	Patient interview
	**Direct cost**		
	Transport cost	Money spent for transport (number of round trips) from home to care providers was compiled.	Patient interview
	Lodging cost	Money spent for food and accommodations for days way from home for consultation were compiled from patient interview.	Patient interview
	Cost of other drugs	Costs of drugs incurred to patients were compiled from the number of prescriptions given to patients and their unit price. Costs incurred in alternative care providers were inquired from patients.	Medical record/patient interview
	Consultations cost	Costs of consultations made in public health facilities were calculated at a cost of $ 0.5 per visit while expenditures at alternative care providers were inquired from patients.	Medical record/patient interview
	Investigation cost.	Costs were compiled from the number of investigation carried our and their unit price.	Medical record
	Cost of hospital admissions	Hospital admission costs were collected for days of admissions at a cost of $ 3.2 per day	Medical record

**Escorts**	**Direct cost**		
	Lodging cost	Expenditures for food and accommodation for numbers of days of stay accompanying patients during consultations were inquired.	Patient interview
	Transport cost	Transport cost was compiled for round trips made from home to care providers.	Patient interview
	**Indirect cost**		
	Income lost for time lost from work travelling with patients from home to public health facilities.	Income lost for hours of travel to public health facilities were estimated at local labour cost ($0.11 per hour for unemployed escorts) and hourly rates derived from their monthly incomes for those who were employed or had regular monthly income.	Patient interview
	Income lost for the time spent accompanying patients during consultations at alternative providers and public health providers.	For unemployed escorts, income lost for number days way from work were estimated using local labour cost of $0.88 per day. Income lost for employed escorts were estimated using rates derived from their monthly income.	Patient interviews

**Public health system**	**Direct costs**		
	Cost of other drugs prescribed to patients for free.	Costs of other drugs were compiled from the number of prescriptions given to patients and their unit price.	Medical record
	Cost of investigations at no cost to patients.	Costs were computed from the number of investigation carried out and their unit price	Medical record
	Cost of hospital admissions at no cost to patients.	Hospital admission costs were computed for days of admission at a cost of $ 3.2 per day	Medical record
	Consultations cost at no cost to patients.	Costs were computed for the number of consultations made in public health facilities at a cost of $ 0.5 per consultation.	Medical record

### Patient cost

Data collected included patient direct costs for TB services and costs incurred to access these services. Direct costs for transport and lodgings were quantified from patient interview. Transport costs were collected from patient-reported costs for round trips and the number of visits made at each care provider. Lodging costs were estimated from patient-reported costs and the number of days of stay for consultation at each care provider. Direct medical costs for the number of consultations made at public health facilities, days of admissions, laboratory investigations carried out and drugs prescribed to patients were quantified from medical records. The costs of health services were determined from the standard fees instituted by the Tigray Regional Health Bureau [16]. Patients who used alternative care providers were interviewed regarding costs they incurred for consultations, drugs and investigations. Patient direct costs of treatment from religious churches (holy water) were not included as these services are provided for free.

Indirect costs were estimated for patients' time lost from work during travel and consultation at care providers. Income loses to patients who had no permanent job and farmers were estimated using local daily wage rate ($ 0.88 per day) for unskilled labour and 28 working days per month. The local daily wage rate for unskilled labour was obtained from the Tigray Regional Labour and Social Affairs Bureau for the year 2005/2006 [17]. For those who were employed or had regular monthly income, daily wage rates derived from their monthly income were used. Income loses for time lost from work due to illnesses and in the processes of patients' consultations at different care providers were not included. Because this study was not aimed to determine the economic impact of TB but costs incurred by patients in seeking TB diagnosis.

### Escort cost

Direct costs encountered by escorts for transport and lodgings were quantified from patient interview. Costs for transport, lodgings and time lost from work were estimated using same assumptions made for patient costs.

### Public health system costs

Health service costs directly attributed to patient diagnosis only were included. Health services given for free to patients were categorised as costs to the public health system. Data on direct costs of consultations, admissions, laboratory investigations and drugs prescribed were quantified from medical records based on the standard charges (unit price) instituted by the Tigray Regional Health Bureau [16] and their frequency of use. Capital costs (buildings) and staff time costs were not estimated primarily because of the difficulty of quantifying the health workers' time commuted to TB service and the high variability of health staffing across public health facilities.

### Data analysis

Data entry and analysis was conducted with SPSS version 14 (SPSS, Inc., Chicago, USA). The various costs collected in Birr were converted into United States Dollars at the official exchange rate of the National Bank of Ethiopia for 2005/2006 of 1$ to Birr 8.8. The mean, median and percentiles of days elapsed on travel and consultation prior to TB diagnosis were calculated. Costs of TB diagnosis were estimated for patients who were screened for TB at the first consulted diagnostic centers and those who were not (mis-diagnosed) in at least the first or more diagnostic centers prior to TB diagnosis. The relations among patients' demographic factors and the different unit cost data were assessed using non-parametric K-independent mean rank test because of the asymmetric nature of cost data.

## Results

A total of 924 newly diagnosed PTB patients (537 smear-positive and 387 smear-negative PTB) were interviewed. Of the total cases, 413 (45%) were female, 576 (52%) were married, 451 (49%) were rural residents, 359 (39%) depended on farming for a livelihood and 430 (47%) were illiterate. The median age was 34 years and family size was 3.

Table 2 summarises time spent by patients and escort during consultation at various care providers from onset of illness to TB diagnosis. A total of 427 patients (46%) had sought care from at least one alternative care provider prior to their first consultation at a public health facility. The median days spent for consultation among patients who had also sought care from alternative care providers was 9 (mean = 14 days). Patients' mean days elapsed at alternative care providers and public health facilities were 5 and 3 days respectively. Forty three percent of patients were accompanied by at least one person to public health facilities. Of the total patients, 108 (12%) were not screened for TB at the first visited diagnostic centres. The median days spent for consultation was lower for patients who were diagnosed in the first visited diagnostic centres than those who were not screened for TB (4 versus 7 days).

**Table 2:** Delays for consultation at care providers by patients and escorts prior to tuberculosis diagnosis in Tigray Region, Ethiopia.

Sources of delays	N (%)	Mean (Median)
**Days elapsed for consultation at care providers:**		
Patients only consulted public health facilities:	497 (54)	3.3 (3)
Consulted alternative providers and public health facilities	427 (46)	14 (9)
Total consultation days at various care providers	924 (100)	8 (4)

**Patients' history of diagnosis of TB at the firs visited diagnostic centres:**		
Patients were diagnosed at the first visited diagnostic centre	816 (88)	8 (4)
Patients were not screened for TB at the first visited diagnostic centres.	108 (12)	12 (7)

The average number of days of hospital admission	924 (100)	3 (0)

The average number of hours travelled by patients to care providers	924 (100)	11 (2)

The average number of escorts per patient	924 (100)	1 (1)

The average number days escorts stayed with patients	924 (100)	6 (1)

The average number of hours travelled by escorts with patients.	924 (100)	17 (0)

Table 3 presents estimated categories of costs of TB diagnosis. The unit median indirect costs for the time spent during consultations and travel at different care providers were $8 (mean = $31) and $0.6 (mean = $2) respectively. The median direct costs spent for diagnosing each patient were: $1.4 (mean = $4) for transport, $5 for lodgings and $9 (median = $18) for medical care. The median total cost spent to diagnose each patient was estimated at $27 (mean = $59). Indirect costs, direct non-medical and direct medical constituted 39%, 16% and 45% of the total cost respectively. The indirect and direct costs comprised 61% and 39% of the total cost spent to diagnose TB patient.

**Table 3:** Types of cost data and categories of costs of TB diagnosis from first consultation to tuberculosis diagnosis.

	**Cost per patient**^1^	
	
Type of costs and categories	Mean	Median (25, 75 percentiles)
**Indirect costs:**		
Income lost from time spent at care providers	31	8 (2.3, 21)
Travel time cost of visiting care providers	2	0.6 (0.2, 2)
**Total indirect cost**	**33**	**10 (3.4, 23)**

**Direct costs**		
**Non-medical**		
Transport cost for visits at public health facilities	4	1.4 (0, 5)
Lodging costs during visits to public health facilities	5	0 (0, 4.3)
**Total non-medical cost**	**9**	**2.3 (0, 9)**
**Medical**		
Cost of other drugs	6	2 (0, 9)
Consultation cost	3	2 (1, 3)
Investigation cost	6	3 (2, 6)
Hospital admissions cost	4	0 (0, 0)
**Total medical cost**	**18**	**9 (4, 19)**
**Direct total**	**26**	**14 (6, 30)**
**Total cost**	**59**	**27 (12, 55)**

^1 ^Figures are computed at Ethiopia National Bank exchange rate of Birr 8.8 to 1 United States Dollar in 2005/06.

Table 4 depicts direct and indirect costs by funding sources to diagnose TB patients. The total median patient cost per patient was $16 (mean = $29), of which the total median indirect and direct costs comprised $6 (mean = $12.2) and $4 (mean = $11) respectively. The total median indirect escort cost per patient incurred by escorts was $2 (mean = $21). The median total escort cost per patient was estimated at $2 (mean = $23.3). Direct medical costs incurred by public health facilities included costs of drugs, investigations, consultations and hospital admissions made prior to TB diagnosis. The median total public health system cost per patient was $3 (mean = $7). Costs attributed to patients, escorts and the public health system respectively constituted 49%, 39% and 12% of the total cost of TB diagnosis.

**Table 4:** Direct and indirect costs incurred by patients, escorts and the public health system prior to tuberculosis diagnosis.

	**Mean (median cost per patient)**^1^			
	
Type of cost data	Patients	Escorts	Public health system	Total cost
**Indirect costs**				
Income lost for the time spent during alternative treatment	6 (1.2)	-	^-^	6 (1.2)
Income lost for the time spent for consultation in public health facilities	6 (2.3)	20.3 (0)	-	31 (8)
Travel time cost of visiting public health facilities	0.8 (0.3)	1 (0.3)	-	2 (0.6)
**Indirect total cost**	**12.2 (6)**	**21 (2)**	**-**	**33 (10)**

**Direct costs**				
**Non-medical**				
Transport cost for visits at public health facilities	2.6 (1.2)	1.6 (0)	-	4 (1.4)
Lodging costs during visits to public health facilities	3.3 (0)	1.3 (0)	-	5 (0)
**Direct total non-medical cost**	**6 (2)**	**3 (0)**	**-**	**9 (2.3)**
**Medical**				
Cost of other drugs	5 (1.6)	-	0.6 (0)	6 (2)
Consultation cost	0.4 (0)	-	1.4 (2)	3 (2)
Investigation cost	4 (1)	-	2.4(2)	6 (3)
Cost of hospital admission	1 (0)	-	0.2 (0)	4 (0)
**Direct total medical**	**11 (4)**	**3 (0)**	**7 (3)**	**18 (9)**

**Total direct cost**	**16 (8)**	**3 (0)**	**7 (3)**	**26 (14)**

**Total cost**	**29 (16)**	**23.4 (2.3)**	**7 (3)**	**59 (27)**

**% to total cost**	**49**	**39**	**12**	**100**

^1 ^Figures are computed at Ethiopia National Bank exchange rate of Birr 8.8 to 1 United States Dollar in 2005/06.

Table 5 depicts the funding sources by patients' characteristics, types of care providers consulted and history of TB diagnosis at diagnostic facilities. The mean unit patient, escort and public health system costs for smear-negative PTB patients were higher than smear-positive patients (Mann-Whitney test, P > 0.01). No significant difference in mean ranks for all categories of unit costs by patients' HIV sero-status (Mann-Whitney test, P < 0.6). Patient who were not screened at the first visited diagnostic centres had a higher patient and escort cost per patient. The mean patient cost faced by each patient was significantly higher for those who were not screened for TB at the first visited diagnostic centres than those who were. Patient cost increased by a factor of 3.6 for those who used alternative care providers as well as mis-diagnosed in 2 or more public health facilities.

**Table 5:** The distribution of patient, escort and public health system costs by respondents' characteristics, pattern of consultation and services given in public health facilities prior to TB diagnosis.

		**Median (mean cost per patient)**^1^			
		
Characteristics	N (%)	Patient cost	Escort cost	Public health system cost	Total cost
**Gender:**					
Female	413 (45)	16.4 (29)	4 (36)	3 (8)	28 (73)
Male	511 (55)	16 (28)	1.4 (13)	3 (6.7)	26 (48)
**Residence**					
Urban	473 (51)	11.5 (28)	0.6 (16)	3 (6)^2^	20.5 (50)
Rural	451 (49)	20 (29.5)	5.2 (33)	2.6 (8)	32 (70)
**Monthly income**					
< 100 Birr	605 (66)	15 (25.5)	2.7 (24)	3 (8)^2^	26.2 (57)
100-200 Birr	177 (19)	19 (36)	2 (10)	2.6 (5)	28.5 (51)
> 200 Birr	142 (15)	19 (33)	0.6 (40)	3 (6)	26.4 (78)
**Did you receive food aid from the government?**					
Yes	231 (25)	11.9 (31.4)	5.2 (13.4)	3 (7.4)	30 (52)
No	693 (75)	15.2 (28)	1.5 (27)	3 (7)	25.6 (61)
**Were you entitled for free medical care?**					
Yes	239 (26)	14 (25)	4.5 (23)	4 (12)^3^	28 (60)
No	347 (38)	16 (26)	2 (32)	2.6 (5)	25.6 (63)
Did not ask	325 (36)	19 (34)	2 (15.5)	2.3 (6)	29 (55)

**Type of tuberculosis disease**					
Smear-positive pulmonary	537 (58)	13 (24)^2^	1.5 (8)^2^	3 (6)^2^	21.6 (38)^2^
Smear-negative pulmonary	387 (42)	23.3 (34.4)	5 (45)	2.6 (8.5)	36 (88)
**HIV sero-status**					
Negative	511 (56)	16.2 (29)	2.4 (26)	3 (7)	27 (62)
Positive	410 (44)	16 (28)	2 (21)	3 (7.3)	26.3 (55)

**Type of care providers consulted prior to TB diagnosis**					
Only public health facilities	497 (54)	10 (21)^3^	2.4 (26)	3 (7)	21 (55)
Both alternative care providers and public health facilities	427 (46)	26 (38)	2 (20)	3 (7)	35 (64)

**Patients' history of diagnosis of TB after** **visiting the first diagnostic facilities**					
They were screened and diagnosed for TB	816 (88)	15 (26)^3^	2 (22)^2^	3 (7)	25 (55)
They were not screened for TB	108 (12)	32 (52)	8 (33)	3.4 (10)	53 (88)

^1 ^Figures are computed at Ethiopia National Bank exchange rate of Birr 8.8 to 1 United States Dollar in 2005/06. ^2 ^P value < 0.05. ^3 ^P-value < 0.01.

## Discussion

The findings reveal many financial burdens to access TB care. Delays are attributed to the complex care pathways followed by patients before diagnosis. Our study showed that delays arising from alternative treatment, health workers' low index of suspicion and inappropriate referral of patients with constitutional signs/symptoms of TB had paramount financial cost implications to patients, their family (escorts) and the public health system.

One of the limitations of the study is under-estimation of the public health system costs as buildings and staff time were not included. Estimating staff time devoted for patient care was difficult because of variations in staffing across health facilities in the referral system. However, the benefit of excluding these costs is that it ensures comparability of findings to other settings where the composition and staffing patterns are different. Second, days of delay from patients' onset of illness to first consultation was not considered in estimating patient cost. This may undermine the financial burden incurred by patients. However, its effect on the overall estimation of costs might not be significant from the fact that most patients seek care at the time when their health status deteriorates and are unable perform daily activities [3]. The costs incurred by patients were comparable to the estimates made by other African studies [4-6].

### Patients' burden in seeking TB care

The economic impact of TB is high in sub-Saharan countries where poverty and HIV is rampant. In this study, most patients were in the productive age group (15 to 50 years) and 48% (445 of 924) were the main sources of income for their family. More than half of patients earn below the poverty line [10] and a quarter of these maintain their daily subsistence through food for work assistance programme. The presence of a TB patient can therefore erode a significant portion of the meagre households' income. Patient direct medical and non-medical expenditures represented 31% and 42% of their median monthly income respectively. The major expenditure for patients was their inability to work in seeking care at alternative and public health providers.

During the median 31 days of illness, patients incurred 125% of their monthly income for medical and non-medical expenses. This can be an insurmountable financial burden to families who were on government food for work programme, and among rural residents who had limited access to public health facilities. The median patient cost per patient incurred to patients was also significantly higher for patients with smear-negative PTB diagnosis than smear-positive PTB. This could be due to atypical presentation of cases with HIV co-infection and prolonged time required to reach smear-negative PTB diagnosis [14]. The NTC diagnostic algorithm requires repeated sputum exams, a trial of treatment with antibiotics and chest x-ray evidence consistent with TB [14]. Patients are therefore required to attend hospitals which are located in district urban centres. Rural patients incurred higher expenses for transport, accommodation and days of stay nearer to hospitals. The significance of economic burden to family members who escort patients has been reported from other Africa countries [4-6]. The costs incurred by escorts constituted a significant portion of the total cost. In this study, the public health system cost was the least component of costs to diagnose a TB patient. Because medical services were provided free to some patients because of their inability pay.

### Strategies to reduce cost of TB diagnosis

We have analysed the appropriateness of care pathways used by patients and its implication on delay and cost of care to reach TB diagnosis. The ideal care pathway for district TB control constitutes: care at one of the diagnostic units (health centre or hospital) and referrals from clinics to diagnostic units or from health centres to hospitals [14,15]. From our analysis, 52% patients sought care using the appropriate district system of referral for TB control programme while the remaining cases sought care from alternative providers prior to their consultation at public health facilities.

Inefficiencies in public health facilities and lack of comprehensive district TB control programme were two main constraints responsible for prolonged delay and increased cost of TB diagnosis. Public health workers failed to suspect and screened them for TB at the time of first contact in diagnostic facilities. This problem arose from health workers' low degree of suspicion of TB and the poor institution of screening policy at district diagnostic units for patients with cough of three weeks duration [14,15]. Currently, TB care is given by one health worker who has received the World Health Organisation TB case management training; however patients are usually screened by untrained health workers who may be unaware of the standard case detection guideline. This finding indicates that screening of patients with suggestive signs/symptoms is not fully instituted across the district referral system. Consequently, patients consulted various public health facilities delaying the time of diagnosis and an unreasonably high cost of TB care. More than a quarter of patients consulted 2 or more diagnostic health facilities within or outside their districts without having the proper TB investigations. The cost of diagnosing TB was alarmingly higher to patients who made their first consultation at private clinics, pharmacies and alternative sources of care.

Improving the efficiency of district TB control programme and the involvement of alternative care providers and the private sector could be important strategies to reduce patient delay to TB diagnosis and its high associated cost. The uses of public health facilities within districts were less costly for patients, their escorts and the public health system in this study. Thus, organising an effective TB care that ensures referral and screening of suspected cases across the district public health system is paramount. This measure requires the institution of a screening policy which minimises mis-diagnosis of patients in district diagnostic facilities; and proper counselling and referral of suspected patients by front-line health facilities. Measures to improve the quality and competence of health providers should be emphasised to minimise undetected cases in public health facilities.

The lack of involvement of the private sector and alternative sources of care in TB control is therefore the second major constraint contributing to patient delay and high cost of care. In this study, 48% of patients consulted private and traditional care providers as an entry point into the public health care system. The involvement of the private and alternative providers in early referral of cases with signs suggestive of TB may reduce delays in diagnosis and avoid unnecessary expenses incurred by patients. Elsewhere, the importance of the use of the private sector in improving case detection has been reported [18,19]. Ethiopia is currently introducing a community-based health service delivery programme, the health extension programme, aimed at the prevention and control of communicable diseases with the involvement of communities. The programme is based on developing a cadre of HEWs who will provide basic curative and preventive health services in every rural community. The integration of TB control activities such as advocacy and health education to communities, early referral of TB suspects into the programme would reduce patient delay to TB diagnosis and cost of care to patients and families.

## Conclusion

The costs incurred by patients in Ethiopia are high, aggravating their poor economic conditions. Delay from alternative treatment, health worker' failure to identify and screen patients with signs/symptoms of TB within district public health facilities were the main causes of the high cost of TB diagnosis. Increasing the involvement of the private sector and traditional sources of care in district TB control, and integrating effective screening and system of referral within district health facilities should be emphasised. Capacity building of public health workers in the district public health system is important to improve timely case detection and to reduce cost of TB diagnosis. All clinicians and health extension workers need to be oriented on the need to screen people with chronic cough with sputum examination, and know the appropriate referral procedures. This demands for intervention studies aimed at identifying methods of integrating a policy of screening of suspected cases at all levels of the district public health system.

## Competing interests

The authors declare no competing interests. The funding source had no role in the study design; collection, analysis or interpretation of data; writing of the report; nor decision to submit the paper for publication.

## Authors' contributions

MMM, RJM, IGT and AG participated in the conception and design of the study. MMM, RJM, IGT, YTK and AG monitored and supervised data collection. MMM, JNN, JDW and MT managed the data, did the analysis and interpretation, and wrote the first and final draft of the manuscript. The manuscript was substantially revised by each author. All authors read and approved the final manuscript.

## Pre-publication history

The pre-publication history for this paper can be accessed here:

http://www.biomedcentral.com/1471-2458/10/173/prepub
